# Syphilis from Accidental Causes

**Published:** 1883-10-20

**Authors:** 


### SYPHILIS FROM ACCIDENTAL CAUSES.

When we consider the numerous ways by which this terrible scourge may be communicated, it should not surprise us to find this enemy often in the midst of the most refined and chaste families, where we would not dare hint at the existence under ordinary circumstances. When we know that the tender infant may have its pure life-blood forever poisoned by the simple touch of an infected nurse; that this child so poisoned, may, with its saliva upon the delicate nipple of its mother, give her also the loathsome disease, and she may transmit to her future offspring the same disease; the thought becomes appalling from its bare possibilities.

Wherever there is the slightest abrasion of the skin, there is a door of entrance for this disease; and wherever the virus from an infected person can be presented, we have the means of propagating the evil.

Public drinking cups, privy seats, money, and the various articles handled by people promiscuously, furnish the media for conveying this disease from the infected to the healthy.

We cite a case in point: A year ago we were consulted by a young gentleman of splendid physique and perfect health, on account of a troublesome *fever blister* on the lower lip which refused to heal. The sore looked angry and leaden in color, was somewhat hardened at its base, and had generally an ugly appearance. I could not trace it to syphilis.

Under all of my efforts it gradually progressed, becoming worse and worse, finally causing the lip to become everted, thickened, and would bleed from time to time. We some how felt that it was syphilitic, but feared epithelioma. I had several eminent medical gentlemen to examine the patient and the universal diagnosis was epithelioma.

It was finally decided to operate, and the patient consenting, I accordingly, with the assistance of Drs. R. H. Cowan, O. A. Crenshaw, and Dr. S. P. Moore (late Surgeon-General C. S. A.) who fully concurred as to diagnosis and the necessity of the operation, excised the diseased mass. The patient suffered, just before and after the operation, with most intense rheumatic pains at night.

Three or four weeks after the operation the patient had some sore throat, and suddenly broke out with a *copper-colored* eruption, or rather, discoloration ; there was some induration about the margin of the wound with a swelling of the submaxillary and sublingual glands.

Being very certain now that syphilis was at the bottom of all the trouble, I again questioned the young man about the possibility of his having drank after any one who might be the subject of constitutional trouble. He then, for the first time, recalled the fact that a week or two before his fever blister troubled him, he had been on a pleasure sail with three other companions in a small boat, and they had a small flask of spirits along, out of which they all drank, one after another, and he having charge of the helm of the boat generally got the last drink ; upon further inquiry, I ascertained that the party who drank just before my patient was suffering from constitutional syphilis at the time.

I immediately prescribed bichloride of mercury and kept him upon it until he was a well man in every particular.

We would advise our professional brethren to look closely for syphilis in many stubborn cases of skin disease, sore throat, ulcers, rheumatism, and other affections of doubtful origin, however light the suspicion of syphilis may appear.—*Southern Clinic.*

